# Immunogenicity and Protective Efficacy of a Non-Living Anthrax Vaccine versus a Live Spore Vaccine with Simultaneous Penicillin-G Treatment in Cattle

**DOI:** 10.3390/vaccines8040595

**Published:** 2020-10-09

**Authors:** Solomon Jauro, Okechukwu C. Ndumnego, Charlotte Ellis, Angela Buys, Wolfgang Beyer, Henriette van Heerden

**Affiliations:** 1 Department of Veterinary Tropical Diseases, Faculty of Veterinary Science, University of Pretoria, Onderstepoort, Pretoria 0110, South Africa; henriette.vanheerden@up.ac.za; 2 Department of Veterinary Microbiology, Faculty of Veterinary Medicine, University of Maiduguri, Maiduguri 600230, Nigeria; 3 Moredun Scientific, Pentlands Science Park, Edinburgh EH26 0PZ, UK; o.ndumnego@moredun-scientific.com; 4 Design Biologixcc, Building 43b CSIR, Brummeria 0184, South Africa; charlotte@designbio.co.za (C.E.); angela@designbio.co.za (A.B.); 5 Department of Livestock Infectiology and Environmental Hygiene, Institute of Animal Science, University of Hohenheim, 70599 Stuttgart, Germany; wolfgang.beyer@uni-hohenheim.de

**Keywords:** anthrax, animal vaccination, non-living vaccine

## Abstract

Sterne live spore vaccine (SLSV) is the current veterinary anthrax vaccine of choice. Unlike the non-living anthrax vaccine (NLAV) prototype, SLSV is incompatible with concurrent antibiotics use in an anthrax outbreak scenario. The NLAV candidates used in this study include a crude recombinant protective antigen (CrPA) and a purified recombinant protective antigen (PrPA) complemented by formalin-inactivated spores and Emulsigen-D^®^/Alhydrogel^®^ adjuvants. Cattle were vaccinated twice (week 0 and 3) with NLAVs plus penicillin-G (Pen-G) treatment and compared to cattle vaccinated twice with SLSV alone and with Pen-G treatment. The immunogenicity was assessed using ELISA against rPA and FIS, toxin neutralisation assay (TNA) and opsonophagocytic assay. The protection was evaluated using an in vivo passive immunisation mouse model. The anti-rPA IgG titres for NLAVs plus Pen-G and SLSV without Pen-G treatment showed a significant increase, whereas the titres for SLSV plus Pen-G were insignificant compared to pre-vaccination values. A similar trend was measured for IgM, IgG1, and IgG2 and TNA titres (NT_50_) showed similar trends to anti-rPA titres across all vaccine groups. The anti-FIS IgG and IgM titres increased significantly for all vaccination groups at week 3 and 5 when compared to week 0. The spore opsonising capacity increased significantly in the NLAV vaccinated groups including Pen-G treatment and the SLSV without Pen-G but much less in the SLSV group with Pen-G treatment. Passive immunization of A/J mice challenged with a lethal dose of 34F2 spores indicated significant protective capacity of antibodies raised in the SLSV and the PrPA + FIS + adjuvants vaccinated and Pen-G treated groups but not for the NLAV with the CrPA + FIS + adjuvants and the SLSV vaccinated and Pen-G treated group. Our findings indicate that the PrPA + FIS + Emulsigen-D^®^/Alhydrogel^®^ vaccine candidate may provide the same level of antibody responses and protective capacity as the SLSV. Advantageously, it can be used concurrently with Penicillin-G in an outbreak situation and as prophylactic treatment in feedlots and valuable breeding stocks.

## 1. Introduction

Anthrax is a bacterial disease caused by the spore-forming bacilli, *Bacillus anthracis*, which infect both animals and humans but is primarily a disease of herbivores [1]. *B. anthracis* causes systemic bacteraemia and toxaemia in its host via virulence factors [2] which are regulated by two plasmids [3]. The pXO1 encodes the tripartite toxin components, namely protective antigen (PA), edema factor (EF), and lethal factor (LF). The EF and LF individually fuse with PA to form anthrax toxins, namely edema toxin (ET) and lethal toxin (LT), respectively [3]. The second plasmid, pXO2, encodes the poly-γ-D-glutamic acid capsule that enables the bacterium to evade host phagocytosis [3].

*B. anthracis* endospores survive in the environment for years and are the source of natural infection through cutaneous, gastrointestinal, or inhalation routes in ruminants [4,5]. During the early phase of acute anthrax infection, endospores which are phagocytosed by macrophages retain their viability and germinate within the macrophages while some may migrate to the regional lymph node and geminate, leading to the production of LT and ET [6]. The ability of the spores to withstand macrophages’ killing ability after phagocytosis enables the bacteria to establish brief intracellular existence before lysing the macrophages to gain access to the host tissue as vegetative cells [6]. The vegetative cells produce the toxins resulting in bacteraemia and subsequently toxaemia as well as oedema in anthrax infection that eventually results in the host death [7,8].

The most effective preexposure prophylactic measure against anthrax as well as curbing the continuity of the disease is vaccination of animals following the appropriate guidelines [5]. In the veterinary field, anthrax is currently controlled using attenuated *B. anthracis* vaccines. Max Sterne developed SLSV by attenuating the culture from an anthrax case in bovine resulting in the avirulent, attenuated *B. anthracis* 34F2 strain without the capsule encoded by pXO2 [9]. The SLSV is a more effective and safer vaccine than Pasteur’s duplex anthrax vaccine, which lacks the pXO1, with reports of the presence of the two plasmids in some of the isolates [5,10,11]. The SLSV has been in use as a veterinary anthrax vaccine of choice in most countries since it was first produced for large-scale immunisation trials in the 1940s [12]. Despite having been considered effective, SLSV has some shortcomings such as residual virulence resulting in the mortality of some vaccinated animals especially goats and llamas [10,11,13], variation in batches during production resulting in inconsistent immune stimulation, and risk of environmental contamination during production or vaccination campaigns. In addition, the live spore vaccine cannot be used simultaneously with antibiotic treatment during a disease outbreak or other scenarios such as the protection of valuable wildlife or feedlots programme where livestock are vaccinated against various diseases and given antibiotics as a prophylactic measure against common diseases [14,15,16,17].

The development of protective antibodies in livestock/animal vaccinated with SLSV has been reported to take 7–10 days [5,14] post-vaccination. Control measures during anthrax outbreaks consist of vaccination of animals immediately after the first case of anthrax, which may result in cessation of further cases [5]. It is advisable to use antibiotics to protect valuable/endangered animals in an anthrax outbreak followed by vaccination with SLSV to ensure future protection after the withdrawal of antibiotics (usually 8-12 days depending on the antibiotics used) [5]. This is because the vaccine is a live spore vaccine which cannot be used simultaneously with antibiotics [5]. In like manner, a withdrawal period of 8-12 days is also observed to allow antibiotics to lapse from the animal’s system before vaccination with SLSV in animals fed with antibiotics-containing feed [16]. Animals suspected to be at risk during anthrax outbreaks are quarantined for 21 days before vaccination [5].

The use of non-living anthrax vaccine (NLAV) candidates would be a novel approach due to the various benefits such as eliminating the danger of handling spores during production, standardising production, avoiding batch variation and environmental contamination, use of improved adjuvants, and simultaneous use with antibiotics treatment [18,19,20]. The human anthrax vaccines, AVP and AVA, are examples of NLAVs that were developed using protective antigen (PA) as the key antigen [21]. These older-generation human anthrax vaccines required numerous boosters to provide sufficient immunity as well as the remnant of lethal factor present with PA harvested from the culture supernatant [22,23,24,25,26]. Thus, recombinant PA (rPA) offers a pure and uncontaminated antigenic component, which can be combined with other *B. anthracis* components to enhance immunity. These components include surface layer proteins [27], exosporium basal layer protein (BxpB also known as ExsF) [2,28], Bacillus collagen-like anthracis (BclA) [29,30,31,32], and/or formalin-inactivated *B. anthracis* spore (FIS) [33,34,35,36,37,38,39]. The idea was to combine rPA which produces anti-toxin antibodies to prevent the production of toxins in vivo with *B. anthracis* spore components to enhance anti-spore antibodies, thus preventing spore germination [17]. In previous studies, goats were vaccinated using rPA, BclA, and FIS adjuvanted with a lipopeptide in a three-step vaccination schedule and protection was shown using either the passive A/J mouse challenge model or lethal virulent *B. anthracis* challenge in goats [36,38]. The immunogenic findings showed that rPA and FIS stimulate a better immune response in the goats compared to BclA and that a two-step vaccination schedule might be sufficient [36,38,40]. A three-step vaccination schedule of rPA, BclA, and FIS was also administered simultaneously with Pen-G to goats and the immune response showed similar antibodies titres against PA and FIS in NLAV plus Pen-G vaccinated group to the animal group vaccinated twice with SLSV [37]. However, the protective efficacy of the antibodies from goats vaccinated with PA, BclA, and FIS and simultaneous treatment with penicillin-G was not investigated. Recently, a two-step vaccination schedule of NLAV candidates (consisting of either purified and crude rPA combined with the FIS and adjuvants) and SLSV administered to cattle showed comparable immunogenicity and protective efficacy induced by the purified rPA + FIS formulation and the standard SLSV [41].

In this study, a two-step vaccination schedule of NLAVs candidates (consisting of either purified or crude rPA combined with the FIS and adjuvants) and SLSV with simultaneous Pen-G treatment as well as SLSV (alone) were administered to cattle, and the immune response and protective potential of the antibodies were determined. The adjuvants used are Emulsigen-D^®^/Alhydrogel^®^ constituting 33% of the vaccine at the ratio of 1:1. Emulsigen-D^®^ is an emulsion formulated using dimethyl-dioctadecyl ammonium bromide (DDA), and Alhydrogel^®^ is a wet suspension of aluminium hydroxide. Emulsigen-D^®^ stimulates a good T-cell response; it also slows the release of antigen from the site of injection and increases the surface area of the antigen. Alhydrogel^®^ enhances antigen presentation cell’s (APC) activity, increases Th2 response and stimulates the secretion of interleukin-1 (IL-1) and interleukin-18 (IL-18) [42,43,44,45,46]. The specific humoral immune responses in the vaccinated cattle were determined using ELISA, toxin neutralisation assay (TNA) and opsonophagocytic assay. The protective efficacy was determined using the passive mouse protection test by in vivo transfer of purified antibodies from the vaccinated cattle and lethal challenge with *B. anthracis* 34F2 spores.

## 2. Materials and Methods

### 2.1. Recombinant Protein Expression and Purification

The rPA83 was expressed and purified as described in Jauro et al. [41]. Briefly, the constructed PA83 coupled to His-tag expressing pStaby1.2 plasmid designated pStaby1.2-PA83.1 in *Escherichia coli* SE1 was induced with 0.3 mM of isopropyl-beta-D-thiogalactopyranoside (IPTG). The protein was purified following the lysis of *E. coli* SE1 by suspending the pellet of the bacteria in lysis buffer (400 mmoL/L NaCl, 50 mmoL/L NaH_2_PO_4,_ and 20 mmoL/L Tris; pH 7.85), followed by three freeze-thawing cycles at 20 °C and 4 °C, respectively, in each cycle. The lysing process also included two cycles of sonication immediately after each freezing and thawing cycle. Each sonication cycle consisted of 20 s of sonication and 10 s without sonication on ice using a probe sonicator (BioLogics, Manassas, VA, USA) three times. Then, the lysate was centrifuged at 2500× *g* for 35 min, and the supernatant was collected. Afterwards, the supernatant received two different treatments to formulate crude and purified rPA. The crude rPA (CrPA) supernatant was treated with Limulus Amoebocyte Lysate (LAL) endosafe endotoxin quantification and removal kit (ThermoFisher Scientific, Rockford, IL, USA). The purified rPA (PrPA) was prepared by purifying the supernatant using Ni^2+^-TED column (Machery-Nagel, Düren, Germany) as instructed by the manufacturer, then filtered using LAL endosafe endotoxin quantification and removal kit (ThermoFisher Scientific, Rockford, IL, USA). SDS PAGE and western blot using 4–20% protein gel (ThermoFisher Scientific, Rockford, IL, USA) were used to confirm the rPA protein, and the yield was quantified with Pierce BCA protein assay kit (ThermoFisher Scientific, Rockford, IL, USA) according to the manufacturer’s protocol. The PrPA procedure was adopted for the rPA83 used in the ELISA. Both CrPA and PrPA concentration for the vaccine formulations were determined as presented in Jauro et al. [41], and PrPA and CrPA vaccines were formulated as presented by Jauro et al. [41].

### 2.2. Formalin-Inactivated Spores (FIS) Preparation

*B. anthracis* 34F2 spores from Onderstepoort Biological Products (OBP), South Africa batch 863 were cultured, inactivated with formalin and tested for sterility as described by Ndumnego et al. [38] and stored in PBS/0.1 g gelatin at −80 °C. The spores used for passive mouse protection test were not inactivated with formalin.

### 2.3. Non-Living Anthrax Vaccines

The NLAVs consisted of the 75 µg crude rPA, 10^8^ FIS, and Emulsigen-D^®^/Alhydrogel^®^ adjuvants in 1 mL dose for each animal as well as 75 µg purified rPA, 10^8^ FIS, and Emulsigen-D^®^/Alhydrogel^®^ adjuvants in a 1 mL dose for each animal was formulated as previously described in Jauro et al. [41].

### 2.4. Animals and Approvals

**Figure 1:**
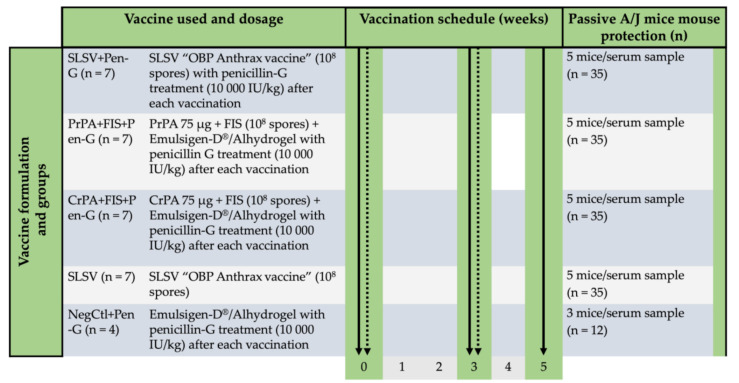
The timeline for two-step vaccination in cattle groups consisting of Sterne live spore vaccine (SLSV) with and without penicillin-G treatment and non-living anthrax vaccines with penicillin-G treatment followed by sera collection and passive mouse protection model. The passive mouse model consisting of A/J mice injected with purified polyclonal IgG from the sera of vaccinated cattle and challenged with *Bacillus anthracis* 34F2 spores. The thick continuous arrows show timepoints for sera collection and the dotted arrows indicate the vaccination timepoints. Sterne live spore vaccine: SLSV; Purified recombinant protective antigen: PrPA; Crude recombinant protective antigen: CrPA; Formalin inactivated *Bacillus anthracis* spores: FIS; Negative control: NegCtl; Penicillin-G: Pen-G.

The cattle study was carried out on a farm following approval under the biosecurity section 20 of the animal disease Act 35 of 1984 by the Director of Animal Health, South Africa (reference nr: 12/11/1/1/6). Based on the approval by the animal ethics committee (protocol number; V118-17 Amendment 1), seven cattle in each group were randomly allocated to the vaccination groups except the negative control groups which comprised of four cattle (Figure 1). The cattle were examined for rPA-reactive antibodies using anti-rPA ELISA before moving to the farm where each animal was treated with 10 mL multivitamins (Kyroligo Reg No. G3087 (Act 36/174)) via intramuscular route and 4 mL Ivermectin (Ivomec injection South African Reg. No. G1142 (Act 36/1947)) subcutaneously. The cattle were fed ad libitum and regularly monitored by a veterinarian.

The in vivo passive mouse protection experiment was conducted in a pathogen-free laboratory of the Onderstepoort Veterinary Animal Research Unit (OVARU) at the University of Pretoria, South Africa, in line with ethical procedures and principles outlined by the University of Pretoria animal ethics committee (protocol number; V118-17 Amendment 1) and section 20 of animal diseases, Act 35 of 1984 (registration number: 12/11/1/1/6(909)). The naïve inbred A/J mice, which lacks the *Hc* gene encoding for complement component 5 (C5) and succumbs to the lethal toxin of *B. anthracis* 34F2 Sterne spores [38] were procured from Jackson Laboratory, USA. The experiment consisted of 5 A/J mice allocated to each serum from vaccinated cattle, whereas the negative control group consisted of 3 mice per serum (Figure 1). For the passive protection test, IgG was purified from the serum of each vaccinated animal with protein G spin column based on the manufacturer’s instruction (NAb™ Protein G Spin Kits, ThermoFisher Scientific, Rockford, IL, USA). ELISA was used to affirm the presence of IgG against PA, and a Pierce BCA protein assay kit (ThermoFisher Scientific, Rockford, IL, USA) was used to quantify the concentration of the IgG. Each A/J mouse was injected with 500 µg of the purified IgG [47] intraperitoneally, and 24 h later each A/J mouse was lethally challenged with 2.16 × 10^5^ *B. anthracis* 34F2 spores (200 µL) spores subcutaneously. The A/J mice were monitored for any clinical signs related to the effect of anthrax toxins for 14 days after the challenge. Death due to anthrax was confirmed by the presence of *B. anthracis* colony morphology from the culture of A/J mouse liver, spleen, and kidney on sheep blood agar after incubation at 37 °C for 24 h followed by the presence of bacilli in Giemsa stained smears. Surviving mice were euthanised after 14 days using isoflurane overdose.

### 2.5. Enzyme-linked Immunosorbent Assay (ELISA) for Serum Immunoglobulins Titre

The anti-PA and anti-FIS ELISAs were used to determine the immunoglobulins titres as previously described by Ndumnego et al. [38]. Briefly, each well was coated with rPA (0.5 μg) or FIS (10^8^) overnight at 4 °C, then washed twice with PBS + 0.05% Tween 20 (PBST) and blocked by incubation for 60 min with PBST + 5% skimmed milk powder (PBSTM) at room temperature. Sera were added to the plates in duplicates starting with the concentration of 1:100 (IgG) and 1:50 (IgM, IgG1 and IgG2) in PBSTM, then 2-fold serially diluted followed by 30 min incubation at room temperature in a shaking incubator at 160 rpm. The secondary antibody goat anti-bovine IgG (ThermoFisher Scientific, Rockford, IL, USA) for IgG detection was added at the concentration of 1:10000 in PTSMP. The secondary antibodies (sheep anti-bovine antibodies) for the detection of IgM, IgG1 and IgG2 were added at the concentration of 1:4000 in PTSMP. After incubation for 30 min at room temperature in a shaking incubator (160 rpm) followed by 5 washes, the plates were developed with 2,2′ azino bis (3 ethylbenzthiazoline-6-sulfonic acid) diammonium salt (Sigma-Aldrich, St. Louis, MO, USA). The absorbance readings were taken on a Biotek Powerwave XS2 plate reader (Boston Laboratory Equipment, Woburn, MA, USA) at 405 nm. The reciprocal of the nearest serum dilution above the cut-off optical density (OD) (mean OD of negative control serum + 3 SD) was considered the endpoint titres of individual sera. Titres of <50 for IgM, IgG1 and IgG2, and IgG were ascribed an arbitrary value of 10. Hyperimmune SLSV vaccinated sera were used as the positive control [40], and the sera from pre-vaccinated animals were used as the negative control.

### 2.6. Toxin Neutralisation Titre (TNA) for Neutralising Antibodies Titre

The in vitro toxin neutralisation ability of vaccinated cattle antibodies was determined as previously described by Ndumnego et al. [38]. Briefly, the TNA was determined with 1.0 × 10^5^ of J774A.1 mouse macrophage cells (ECACC cat no 91051511) per well followed by overnight incubation at 37 °C and 5% CO_2_. Then sera from vaccinated animals were 2-fold serially diluted in duplicates starting from the dilution of 1:50. Then the macrophages were exposed to anthrax lethal toxin (PA: 500 ng/mL and LF: 400 ng/mL (List Biological Laboratories Inc., Campbell, CA, USA) in the presence of antibodies. Afterwards, 5 mg/mL MTT (3-(4,5 dimethylthiazol-2-yl)-2,5-diphenyltetrazolium bromide (Invitrogen, Carlsbad, CA, USA) was added for signalling and developed with formazan crystal dye after adding acidified dye. Finally, a Biotek power wave XS2 plate reader was used to read the plates’ absorbance at 540 nm. The neutralisation titres at which the complementary serum dilution enables J774A.1 macrophage cell’s survival yielded 50% neutralisation using Gen5 data analysis software (Biotek Instruments, Winooski, VT, USA) with designated neutralisation titres (NT_50_).

### 2.7. Opsonophagocytic Assay

The opsonophagocytic potential of induced antibodies was evaluated on RAW 264.7 macrophage cells as previously carried out by Welkos et al. [17], with few modifications. Briefly, heat-activated, refractile ungerminated *B. anthracis* spores (2.6 × 10^9^ spores/mL) were pre-incubated with 10-fold serial dilutions of the immune sera and sera from the negative control (NegCtl), for 30 min at 4 °C and then added to RAW 264.7 macrophage cells (5 × 10^5^ cells/well) and incubated for 45 min at 37 °C in 5% CO_2_. The macrophage cells were washed with sterile PBS (pH 7.4 ± 1) and incubated with DMEM containing 10% FBS and 10 μg/mL gentamicin at 37 °C in 5% CO_2_ for 30 min to remove vegetative bacilli. Subsequently, the macrophage cells were washed with sterile ice-cold PBS and incubated for 5 min in 100 μL 0.1% Triton^®^ X100 (ThermoFisher Scientific, Rockford, IL, USA) to lyse the macrophages and were plated on LB media to count viable cfu/ml. Data are presented as percentage spore uptake by the macrophage cells. Sera from pre-vaccination screening were used as a negative control, whereas the group vaccinated with SLSV alone from this study was used as the positive control.

### 2.8. Statistical Analysis

Gen 5 data analysis software (Biotek Instruments, Winooski, USA) was used to generate 4-parameter logistic curves for the ELISA and TNA titres. The data collected were log-transformed using GraphPad prism version 8.3.0 software. The antibody titres between groups at different time points on ELISA and TNA were compared using unpaired Student *t*-test with a two-tailed *p*-value and Kruskal–Wallis test followed by Dunn’s multiple comparisons test with adjusted *p*-value. The mean survival time of the challenged A/J mice was plotted using the Kaplan–Meier survival curve. The log-rank (Mantel–Cox) test was used to compare survival between different vaccination groups. All graphical elucidations and the analysis were done using GraphPad Prism version 8.3.0 software.

## 3. Results

### 3.1. Humoral IgG Titre

**Figure 2:**
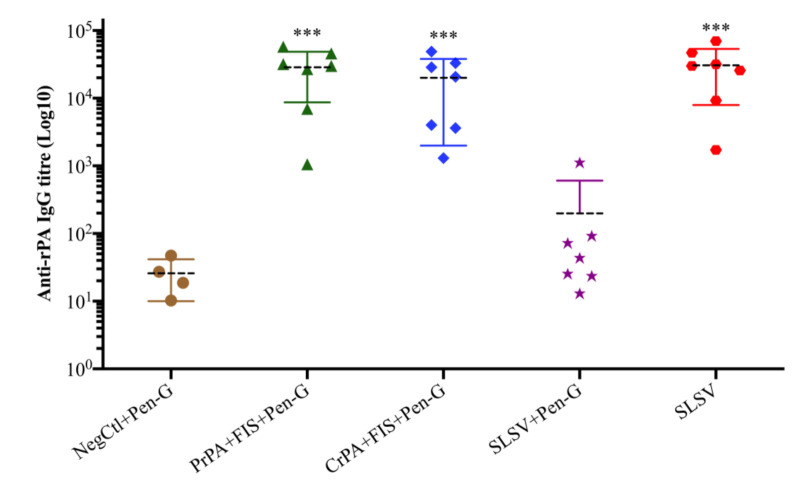
Anti-recombinant protective antigen (rPA) IgG-titres at week 5 in cattle. The cattle were vaccinated twice at weeks 0 and 3 with PrPA + FIS + Emulsigen-D^®^/Alhydrogel^®^ plus Pen G (*n* = 7), CrPA + FIS + Emulsigen-D^®^/Alhydrogel^®^ plus Pen G (*n* = 7), Sterne live spore vaccine (SLSV) plus Pen-G (*n* = 7), SLSV (*n* = 7) and NegCtl (Emulsigen-D^®^/Alhydrogel^®^ plus Pen-G) (*n* = 4). The IgG titres of each group were compared to the respective titres at week 0 before vaccination (*** *p* < 0.001, ** *p* < 0.01, * *p* ≤ 0.05). Purified recombinant protective antigen: PrPA; Crude recombinant protective antigen: CrPA; Formalin inactivated *Bacillus anthracis* spores: FIS; Penicillin-G: Pen-G; Sterne live spore vaccine: SLSV; Negative control: NegCtl.

**Figure 3:**
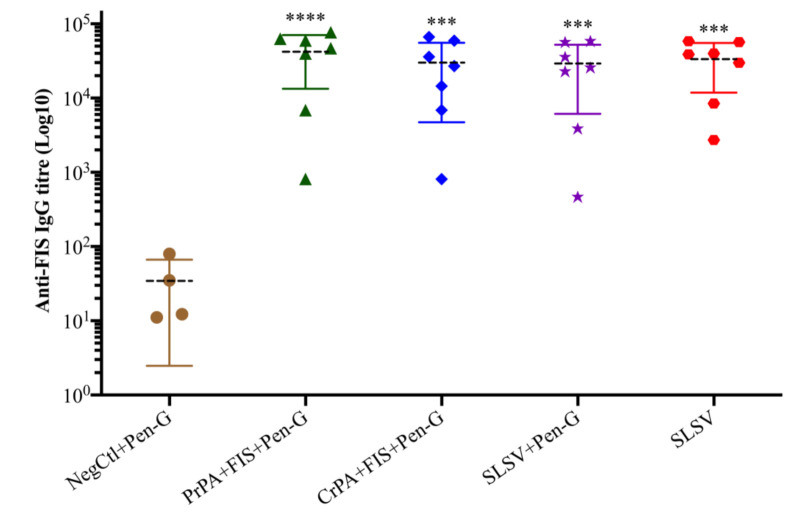
Anti-formalin inactivated *Bacillus anthracis* 34F2 spores (FIS) IgG-titres at week 5 in cattle. The cattle were vaccinated twice at weeks 0 and 3 with PrPA + FIS + Emulsigen-D^®^/Alhydrogel^®^ plus Pen G (*n* = 7), CrPA + FIS + Emulsigen-D^®^/Alhydrogel^®^ plus Pen G (*n* = 7), Sterne live spore vaccine (SLSV) plus Pen-G (*n* = 7), SLSV (*n* = 7) and NegCtl (Emulsigen-D^®^/Alhydrogel^®^ plus Pen-G) (*n* = 4). The IgG titres of each group were compared to the respective titres at week 0 before vaccination (**** *p* < 0.0001, *** *p* < 0.001, ** *p* < 0.01, * *p* ≤ 0.05). Purified recombinant protective antigen: PrPA; Crude recombinant protective antigen: CrPA; Formalin inactivated *Bacillus anthracis* spores: FIS; Penicillin-G: Pen-G; Sterne live spore vaccine: SLSV; Negative control: NegCtl.

Both the PrPA + FIS + Emulsigen-D^®^/Alhydrogel^®^ plus Pen-G and CrPA + FIS + Emulsigen-D^®^/Alhydrogel^®^ plus Pen-G stimulated a significant IgG response against rPA, at week 3 (three weeks after the first vaccination), whereas the anti-rPA IgG titres were insignificant among the animal groups that were vaccinated with SLSV + Pen-G as well as SLSV alone at week 3 (Table S1). At week 5 (two weeks after the second vaccination), the IgG titres against rPA increased significantly for PrPA + FIS + Emulsigen-D^®^/Alhydrogel^®^ plus Pen-G, CrPA + FIS + Emulsigen-D^®^/Alhydrogel^®^ plus Pen-G and SLSV vaccinated groups, while SLSV + Pen-G titres increased but not significantly (Table S1, Figure 2). In addition, the anti-FIS IgG titres for PrPA + FIS + Emulsigen-D^®^/Alhydrogel^®^ plus Pen-G, CrPA + FIS + Emulsigen-D^®^/Alhydrogel^®^ plus Pen-G, SLSV plus Pen-G and SLSV at week 3 and week 5 increased significantly (Table S1, Figure 3). Both anti-rPA and anti-FIS titres were compared to pre-vaccination titres (week 0).

### 3.2. Humoral IgM and IgG Isotypes Titre

**Figure 4:**
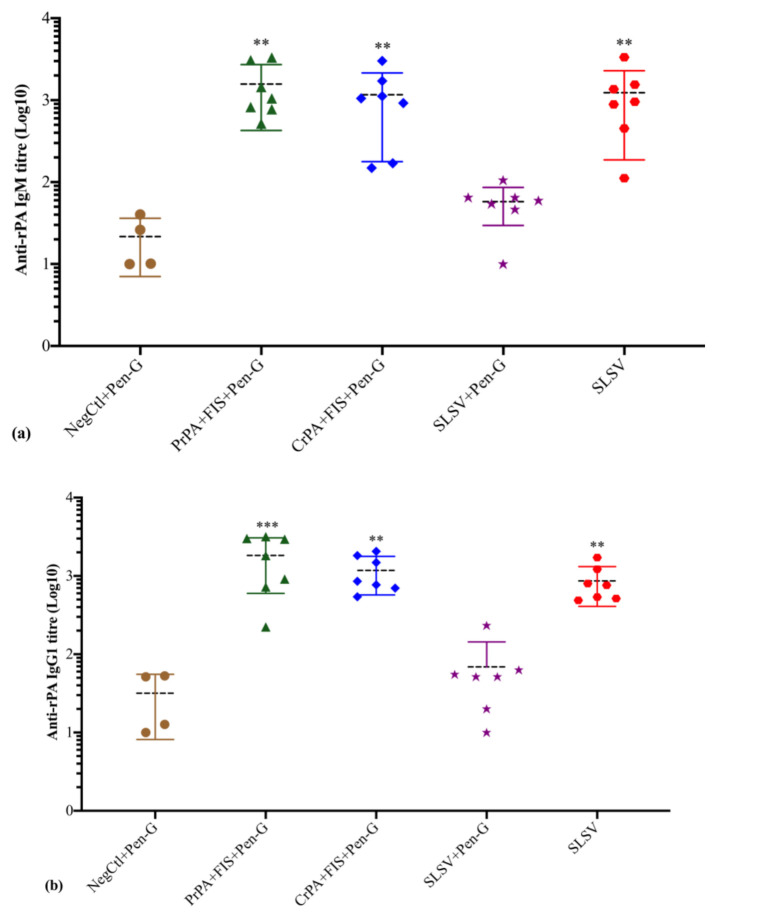

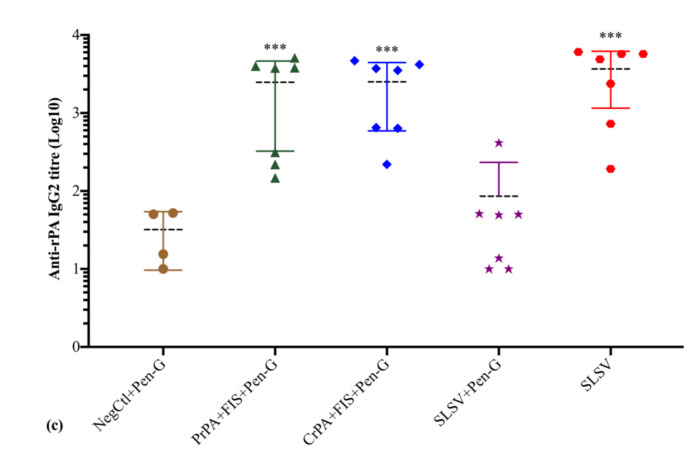
Anti-recombinant protective antigen (rPA) IgM and IgG isotypes (IgG1 and IgG2) titres at week 5 in cattle. The cattle were vaccinated twice at weeks 0 and 3 with PrPA + FIS + Emulsigen-D^®^/Alhydrogel^®^ plus Pen G (*n* = 7), CrPA + FIS + Emulsigen-D^®^/Alhydrogel^®^ plus Pen G (*n* = 7), Sterne live spore vaccine (SLSV) plus Pen-G (*n* = 7), SLSV (*n* = 7) and NegCtl (Emulsigen-D^®^/Alhydrogel^®^ plus Pen G) (*n* = 4). The IgG titres of each group were compared to the respective titres at week 0 before vaccination (*** *p* < 0.001, ** *p* < 0.01, * *p* ≤ 0.05). (**a**) anti-FIS IgM titres, (**b**) anti-FIS IgG1 titres and (**c**) anti-FIS IgG2 titres. Purified recombinant protective antigen: PrPA; Crude recombinant protective antigen: CrPA; Formalin inactivated *Bacillus anthracis* spores: FIS; Penicillin-G: Pen-G; Sterne live spore vaccine: SLSV; Negative control: NegCtl.

The anti-rPA IgM titres increased significantly at both week 3 and 5 among PrPA + FIS + Emulsigen-D^®^/Alhydrogel^®^ plus Pen-G, CrPA + FIS + Emulsigen-D^®^/Alhydrogel^®^ plus Pen-G and SLSV vaccinated groups. The anti-rPA IgM response in the group vaccinated with SLSV + Pen-G revealed insignificant titres at weeks 3 and 5. The levels of anti-rPA IgG1 titres showed a significant increase for PrPA + FIS + Emulsigen-D^®^/Alhydrogel^®^ plus Pen-G, CrPA + FIS + Emulsigen-D^®^/Alhydrogel^®^ plus Pen-G and SLSV after the first (week 3) and second (week 5) vaccination. The anti-rPA IgG2 titres level displayed a significant response at week 3 and highly significant response at week 5 for PrPA + FIS + Emulsigen-D^®^/Alhydrogel^®^ plus Pen-G, CrPA + FIS + Emulsigen-D^®^/Alhydrogel^®^ plus Pen-G and SLSV. However, the group vaccinated with SLSV plus Pen-G showed insignificant anti-rPA titres against IgG1 and IgG2 at weeks 3 and 5 (Table S2, Figure 4). All titres were compared to pre-vaccination (week 0) anti-rPA IgM, IgG1 and IgG2 titres. However, the NegCtl showed insignificant IgM and IgG isotypes response against rPA among all vaccine groups (Table S2, Figure 4).

**Figure 5:**
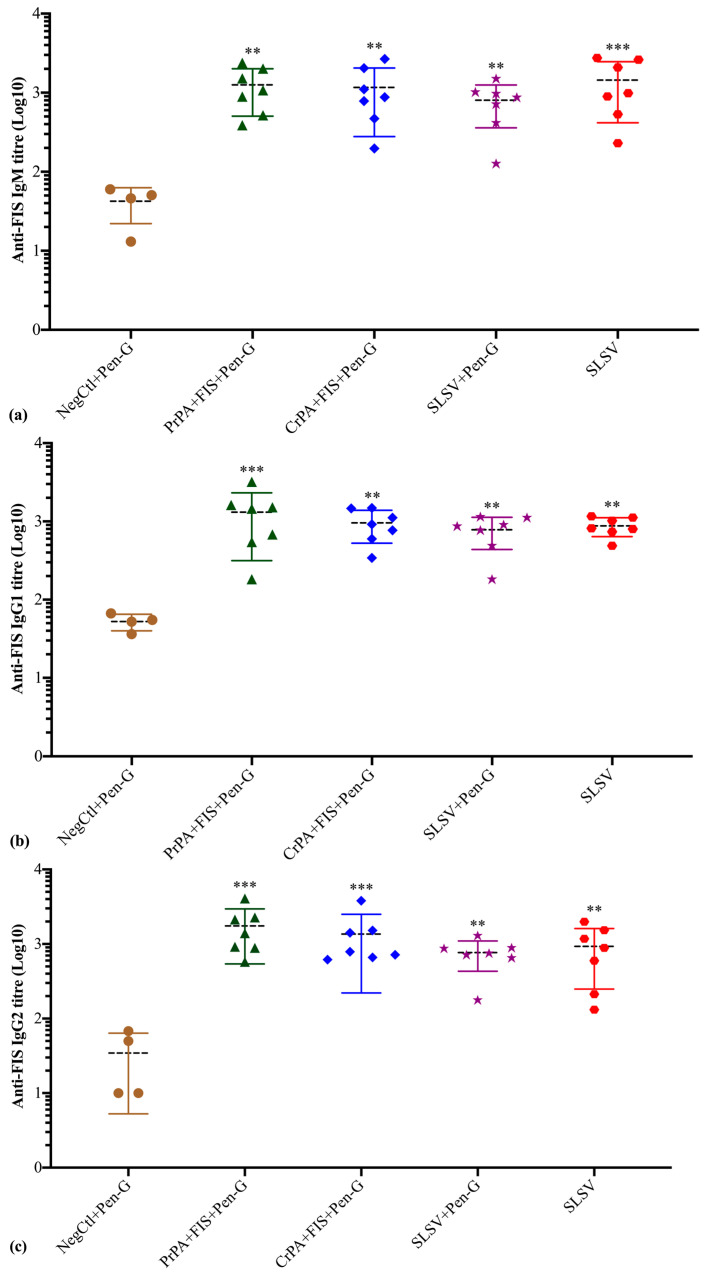
Anti-formalin inactivated *Bacillus anthracis* 34F2 spores (FIS) IgM and IgG isotypes (IgG1 and IgG2) titres at week 5 in cattle. The cattle were vaccinated twice at weeks 0 and 3 with PrPA + FIS + Emulsigen-D^®^/Alhydrogel^®^ plus Pen G (*n* = 7), CrPA + FIS + Emulsigen-D^®^/Alhydrogel^®^ plus Pen G (*n* = 7), Sterne live spore vaccine (SLSV) plus Pen-G (*n* = 7), SLSV (*n*=7) and NegCtl (Emulsigen-D^®^/Alhydrogel^®^ plus Pen G) (*n* = 4). The IgG titres of each group were compared to the respective titres at week 0 before vaccination (*** *p* < 0.001, ** *p* < 0.01, * *p* ≤ 0.05). (**a**) anti-FIS IgM titres, (**b**) anti-FIS IgG1 titres and (**c**) anti-FIS IgG2 titres. Purified recombinant protective antigen: PrPA; Crude recombinant protective antigen: CrPA; Formalin inactivated *Bacillus anthracis* spores: FIS; Penicillin-G: Pen-G; Sterne live spore vaccine: SLSV; Negative control: NegCtl.

The anti-FIS IgM response revealed a significant increase in titres at week 3 and week 5 among the cattle groups vaccinated with PrPA + FIS + Emulsigen-D^®^/Alhydrogel^®^ plus Pen-G, CrPA + FIS + Emulsigen-D^®^/Alhydrogel^®^ plus Pen-G, SLSV plus Pen-G and SLSV when compared to week 0 titres (Table S2, Figure 5). The anti-FIS IgG1 isotype response was significant among cattle groups vaccinated with PrPA + FIS + Emulsigen-D^®^/Alhydrogel^®^ plus Pen-G, CrPA + FIS + Emulsigen-D^®^/Alhydrogel^®^ plus Pen-G, SLSV plus Pen-G and SLSV after the first vaccination (Table S2). The anti-FIS IgG1 titres were higher and more significant at week 5 across all vaccination groups when compared to pre-vaccination titres (Table S2, Figure 5). The IgG2 isotype response against FIS showed a significant response at week 3 against PrPA + FIS + Emulsigen-D^®^/Alhydrogel^®^ plus Pen-G, CrPA + FIS + Emulsigen-D^®^/Alhydrogel^®^ plus Pen-G, SLSV plus Pen-G and SLSV (Table S2). The anti-FIS IgG2 isotype revealed higher titres that are highly significant at week 5 among all vaccine groups (Table S2, Figure 5). All anti-FIS IgG2 titres at weeks 3 and 5 were compared to pre-vaccination IgG2 titres. The NegCtl showed insignificant IgM and IgG isotypes response against FIS throughout the study (Table S2, Figure 5).

### 3.3. Toxin Neutralisation Antibodies Titre (NT_50_)

**Figure 6:**
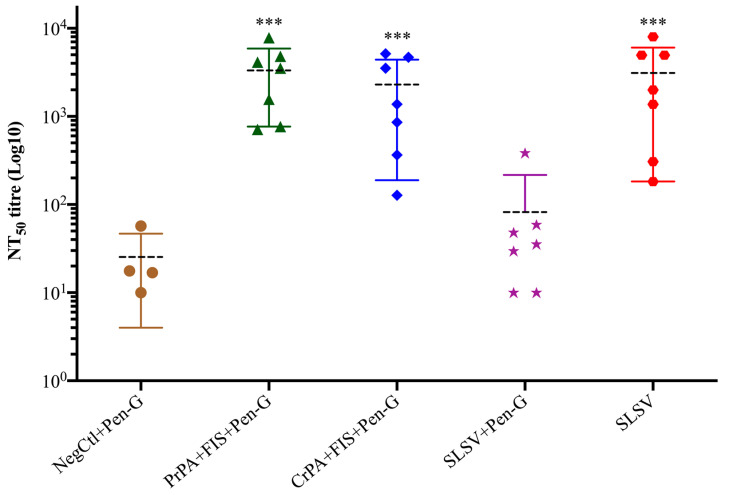
Lethal toxin neutralising titres at week 5 in vaccinated cattle. The cattle were vaccinated twice (weeks 0 and 3) with PrPA + FIS + Emulsigen-D^®^/Alhydrogel^®^ plus Pen-G (*n* = 7), CrPA + FIS + Emulsigen-D^®^/Alhydrogel^®^ plus Pen-G (*n* = 7), with SLSV plus Pen-G (*n* = 7), and SLSV (*n* = 7), and the naïve control consisting of Emulsigen-D^®^/Alhydrogel^®^ plus Pen-G (NegCtl) (*n* = 4) received the vaccine diluent and Pen-G on weeks 0 and 3. NT_50_ of each group were compared to the respective titres at week 0 before vaccination (*** *p* < 0.001, ** *p* < 0.01, * *p* ≤ 0.05). Purified recombinant protective antigen: PrPA; Crude recombinant protective antigen: CrPA; Formalin inactivated *Bacillus anthracis* spores: FIS; Penicillin-G: Pen-G; Sterne live spore vaccine: SLSV; Negative control: NegCtl.

The neutralising antibody titres (NT_50_) were low after the first vaccination at week 3 without any significant increase for all vaccine groups, but the NT_50_ increased significantly for PrPA + FIS + Emulsigen-D^®^/Alhydrogel^®^ plus Pen-G, CrPA + FIS + Emulsigen-D^®^/Alhydrogel^®^plus Pen-G and SLSV vaccinated groups after the second vaccination at week 5. However, there was no significant increase observed in NT_50_ titres among the vaccine groups immunised with SLSV and treated with Pen-G after the first and second vaccination (Table S1, Figure 6).

### 3.4. Macrophages Spore Uptake Induced by Opsonising Antibodies

**Figure 7:**
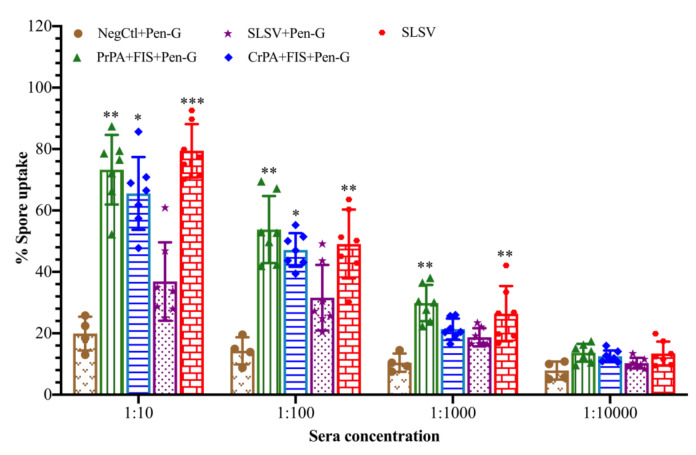
*Bacillus anthracis* 34F2 spores phagocytosis by RAW 267.7 macrophages with varying dilutions of sera from cattle groups vaccinated with PrPA + FIS + Emulsigen-D^®^/Alhydrogel^®^ plus Pen-G, CrPA + FIS + Emulsigen-D^®^/Alhydrogel^®^ plus Pen-G, SLSV plus Pen-G, SLSV alone groups and NegCtl groups (Emulsigen-D^®^/Alhydrogel^®^ plus Pen-G). The mean value of spore uptake is presented in the form of bar charts with the standard deviations. The significant values between groups are presented as *** *p* < 0.001, ** *p* < 0.01, * *p* ≤ 0.05. Purified recombinant protective antigen: PrPA; Crude recombinant protective antigen: CrPA; Formalin inactivated *Bacillus anthracis* spores: FIS; Penicillin-G: Pen-G; Sterne live spore vaccine: SLSV; Negative control: NegCtl (Emulsigen-D^®^/Alhydrogel^®^ plus Pen-G).

The ability of the vaccine-induced antibodies collected at week 5 to opsonise and enhance phagocytosis of *B. anthracis* spores by macrophages (RAW264.7) was measured. The highest level of spore uptake by the macrophage at the sera dilution of 1:10 was 79% for SLSV, 73% for PrPA + FIS + Emulsigen-D^®^/Alhydrogel^®^ plus Pen-G and 61% for CrPA + FIS + Emulsigen-D^®^/Alhydrogel^®^ plus Pen-G, whereas an insignificant level of spore uptake by macrophages was recorded for SLSV + Pen-G (37%) and NegCtl (20%) (Figure 7). A significant spore uptake by macrophages among cattle groups vaccinated with PrPA + FIS + Emulsigen-D^®^/Alhydrogel^®^ plus Pen-G, and SLSV in dilutions of 1:10, 1:100, 1:1000 was observed, whereas CrPA + FIS + Emulsigen-D^®^/Alhydrogel^®^ plus Pen-G vaccine group showed significant spore uptake at the sera dilution of 1:10 and 1:100 only. The macrophages spore uptake across all vaccination groups was compared to the level of spore uptake by negative control sera.

### 3.5. Protection Conferred on A/J Mice by Antibodies from Cattle Immune Sera

**Figure 8:**
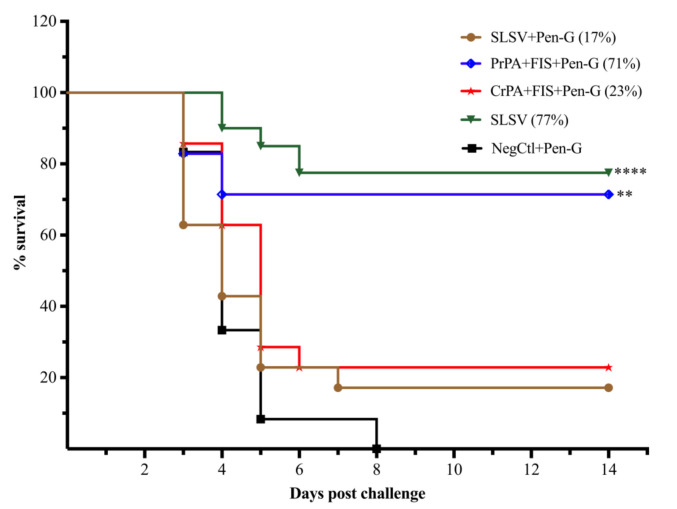
Mice survival curve following passive in vivo transfer of purified IgG from sera from cattle to A/J mice. The A/J mice were lethally challenged with approximately 2.16 × 10^5^ *B. anthracis* 34F2 spores. The polyclonal IgG are from sera of cattle vaccinated twice (week 0 and 3) with either PrPA + FIS + Emulsigen-D^®^/Alhydrogel^®^ plus Pen-G, CrPA + FIS + Emulsigen-D^®^/Alhydrogel^®^ plus Pen-G, SLSV plus Pen-G, SLSV alone groups and NegCtl groups (Emulsigen-D^®^/Alhydrogel^®^ plus Pen-G). The survival rate in Log-rank (Mantel–Cox) test was compared to NegCtl group. The significant values between groups are presented as **** *p* < 0.0001, *** *p* < 0.001, ** *p* < 0.01. Purified recombinant protective antigen: PrPA; Crude recombinant protective antigen: CrPA; Formalin inactivated *Bacillus anthracis* spores: FIS; Penicillin-G: Pen-G; Sterne live spore vaccine: SLSV; Negative control: NegCtl (Emulsigen-D^®^/Alhydrogel^®^ plus Pen-G).

The protection of A/J mice passively immunised with the purified polyclonal antibodies from SLSV plus Pen-G and CrPA + FIS + Emulsigen-D^®^/Alhydrogel^®^ plus Pen-G cattle groups was insignificant with 17% (6/35) and 23% (8/35) of the A/J mice surviving lethal challenge, respectively (Figure 8). In contrast, the A/J mice immunised with the polyclonal IgG from cattle groups vaccinated with SLSV and PrPA + FIS + Emulsigen-D^®^/Alhydrogel^®^ plus Pen-G showed a significant level of 77% (27/35) and 71% (25/35) protection of the immunised A/J mice, respectively (Figure 8).

## 4. Discussion

SLSV is the sole vaccine available for control of anthrax in animals [12]. Yet, it has shortcomings which include residual virulence in some ruminant species such as goats and llamas [11,13]. It is also incompatible with antibiotic prophylaxis. Webster [16] reported that SLSV failed to stimulate protective antibodies in guinea pigs in the presence of antibiotics treatment. The latter implies that SLSV cannot be used concurrently with antibiotic treatment in anthrax outbreak situations for the protection of livestock and endangered wildlife [15,16], or in feedlots where livestock especially cattle are treated and vaccinated against common diseases as a prophylactic measure [48]. In this study, both CrPA + FIS + Emulsigen-D^®^/Alhydrogel^®^ plus Pen-G and PrPA + FIS + Emulsigen-D^®^/Alhydrogel^®^ plus Pen-G vaccinated cattle groups and SLSV alone cattle group induced significant anti-rPA antibody response two weeks after the second vaccination (week 5), whereas the cattle vaccinated twice with SLSV with simultaneous penicillin-G treatment were unable to induce adequate anti-rPA antibody titres (Table S1, Figure 2). Similar trends were observed for lethal toxin neutralising antibodies titres (Table S1, Figure 6). The pattern of response was similar to anti-FIS antibody titres except that it recorded significant antibody titres in the group vaccinated with SLSV + Pen-G also (Table S1, Figure 3). Our findings indicate that the presence of penicillin-G may inhibit the PA-specific immune response without significant effects on the immunogenicity of *B. anthracis* spore. Immune data from this study showed a similar pattern to that observed in goats vaccinated thrice with either rPA + BclA + FIS + lipopeptide plus Pen-G, SLSV or SLSV + Pen-G by Ndumnego [37]. The authors reported the neutralising antibodies, and the IgG against PA was higher in two of the five goats vaccinated with rPa + BclA + FIS + lipopeptide plus Pen-G, but due to the low number of animals in each vaccination group, the results were not significant. In our study, the IgM and IgG isotype response has shown sudden increase even though it was dominated by IgM and IgG1 at week 3. The IgM and IgG isotypes maintained the level of response until the fifth week when the level of IgG2 dominates week 5 across all the vaccine groups except SLSV plus Pen-G (Table S2). The stimulation of both IgG1 and IgG2 implies a balance between Th1 and Th2 response.

In this study, we used PA, which is the major component of *B. anthracis* tripartite proteins encoded by pXO1 and the principal immunogenic component. PA stimulates an early immune response against anthrax toxins; however, studies have shown that the immunity stimulated by PA is short-lived [22,26]. To stimulate a protective and lasting immune response, several studies coupled PA with other non-living components of *B. anthracis* such as BclA, BxpB, or even the whole FIS to intensify the immune response. The majority of these studies were conducted in laboratory rodents except the studies conducted by Ndumnego et al. [38] and Koehler et al. [36] which reported the immunogenicity of NLAV formulation comprising of rPa + BclA + FIS + lipopeptide in goats vaccinated thrice. In these studies, PA and FIS stimulated better immunity than BclA with results indicating that two vaccinations might be sufficient to render protection. The two-step vaccination schedule was suggested based on antibody titres after second vaccination in rPA + BclA + FIS + lipopeptide in vaccinated goats, which did not differ significantly from the antibodies titres after the third vaccination and were similar to goats vaccinated twice with SLSV [36,37,38]. The two-step vaccination schedule was confirmed in cattle using the CrPA + FIS + Emulsigen-D^®^/Alhydrogel^®^ plus Pen-G and PrPA + FIS + Emulsigen-D^®^/Alhydrogel^®^ plus Pen-G vaccine formulations which stimulated a significant immune response with the latter significantly protecting A/J mice from the lethal challenge [41]. The immunogenic and protective ability of PrPA + FIS + Emulsigen-D^®^/Alhydrogel^®^ plus Pen-G vaccine is thus comparable with SLSV in cattle [41] and consequently resulted in the current study to test the simultaneous use of antibiotic treatment with NLAVs.

The level of antibodies against rPA and FIS as well as neutralising antibodies response in the two-step cattle vaccination schedule with NLAVs plus Pen-G revealed similar immunogenic trends as seen in two-step cattle SLSV vaccination. The levels of antibody titres for cattle groups vaccinated with SLSV plus Pen-G were inhibited and differed significantly from NLAVs + Emulsigen-D^®^/Alhydrogel^®^ plus Pen-G and SLSV. The polyclonal IgG from PrPA + FIS + Emulsigen-D^®^/Alhydrogel^®^ plus Pen-G vaccine group demonstrated a promising level of protection by saving 71% of the A/J mice. This is comparable with the level of protection conferred to A/J mice (77%) by polyclonal IgG from the SLSV vaccine group. However, low level of protection of A/J mice (17%) was recorded by SLSV plus Pen-G polyclonal IgG against *B. anthracis* 34F2 spore challenge. Our finding revealed that the presence of antibiotic treatment in the vaccination schedule influenced the inhibition of the immunogenic ability of SLSV as previously reported by Webster [16]. The polyclonal IgG from CrPA + FIS + Emulsigen-D^®^/Alhydrogel^®^ plus Pen-G vaccinated group afforded 23% protection of A/J mice which is low considering the antibody responses in this group (Table S1, Figure 3 and Figure 4). This is similar to the polyclonal IgG from CrPA + FIS + Emulsigen-D^®^/Alhydrogel^®^ plus Pen-G vaccinated cattle that also provided only 20% protection to A/J mice in the study of Jauro et al. [41]. Based on our finding, this may be associated with the method used to obtain the crude rPA which filters out only the *E. coli* endotoxin and allows other proteins in the final products [49]. In addition, it has been indicated by Welkos et al. [6] that anti-PA antibodies play an important role in opsonising spores and, hence, increase the spore uptake by macrophages. Therefore, the anti-PA antibodies raised against the crude rPA could be less effective in this respect; this would not be visible in ELISA or TNA but only in the opsonophagocytic test and finally, in the passive mouse protection test.

Macrophages are primary effector cells of host innate immune response that fight bacterial infection [50]. The spore opsonising ability of antibodies from vaccinated cattle was evaluated in our study. Our findings revealed that NLAVs with simultaneous penicillin-G treatment and SLSV without penicillin-G treatment induced the production of opsonising antibodies which were able to prevent 73% (group vaccinated with PrPA + FIS + Emulsigen-D^®^/Alhydrogel^®^ plus Pen-G), 63% (group vaccinated with CrPA + FIS + Emulsigen-D^®^/Alhydrogel^®^ plus Pen-G) and 79% (group vaccinated with SLSV) of the spores from escaping phagocytosis by macrophages at the serum dilution of 1:10. Our findings are similar to the results of Jauro et al. [41], which reported spore opsonising ability of antibodies from the NLVAs tested in cattle without Pen-G were able prevent 75% (group vaccinated with PrPA + FIS + Emulsigen-D^®^/Alhydrogel^®^) and 66% (group vaccinated with CrPA + FIS + Emulsigen-D^®^/Alhydrogel^®^) of the spores from dodging phagocytosis by macrophage at the serum dilution of 1:10. Our data support the important role of opsonising antibodies in the immune response against infection with spores of *B. anthracis*. Besides the test for antibodies capable of lethal toxin neutralisation, commonly accepted as a correlate for the protective immune response against infection with *B. anthracis*, testing the spore opsonising capacity of antibodies could substantially add to our methodological repertoire when testing vaccine candidates comprising cellular antigens as well. Further studies will be necessary to identify which IgG isotype that is primarily responsible for engaging FCγRs on the macrophages and influencing spore phagocytosis following vaccination with PA and FIS.

## 5. Conclusions

Our study indicates the ability of a non-living vaccine candidate (NLAV) (PrPA + FIS + Emulsigen-D^®^/Alhydrogel^®^) to stimulate a protective immune response against both *B. anthracis* PA and spores under the conditions of concomitant antibiotic treatment in cattle. More importantly, the level of immune responses witnessed was comparable with those of the living spore (34F2) immunised group after two vaccinations. Our finding revealed the feasibility of adopting NLAV using a purified rPA and FIS as an alternative animal anthrax vaccine which can offer the benefit of concurrent use with antibiotic treatment in the phase of an infectious disease outbreak. Such an approach can significantly reduce economic losses should animals incubate a disease that requires antimicrobial treatment, and vaccination against anthrax is desired at the same time. The novelty of our approach is in line with the recent FDA approval to simultaneously used AVA PA-based human vaccine with antimicrobial treatment in an anthrax outbreak or any disease that requires antimicrobial treatment [51]. The purified antibodies from animals vaccinated with NLAV have demonstrated significant passive protection ability in A/J mice which is comparable with the level of protection conferred by antibodies from SLSV sera. Future work will be initiated to investigate the cellular immune response and to determine the duration of the immunity.

## Supplementary Materials

The following are available online at https://www.mdpi.com/2076-393X/8/4/595/s1, Table S1. The Anti-recombinant protective antigen (rPA) and anti-formalin inactivated spores (FIS) IgG titres (log10) in cattle vaccinated at weeks 0 and 3 and measured at weeks 0, 3, and 5 (with means and standard deviations). Cattle were vaccinated twice (weeks 0 and 3) with purified rPA (PrPA) + FIS + Pen-G (penicillin-G), crude rPA (CrPA) + FIS + Pen-G, SLSV + FIS + Pen-G, SLSV and NegCtl + Pen G (vaccinated with Emulsigen-D^®^/Alhydrogel^®^ plus Pen-G), Table S2. The anti-recombinant protective antigen (rPA) and anti-formalin inactivate *Bacillus anthracis 34F2* spores (FIS) titres (log10) of different isotypes in vaccinated cattle (with means and standard deviations) of immunoglobulin isotypes titre of vaccinated cattle measured at weeks 0, 3 (two weeks after first vaccination) and 5 (two weeks after second vaccination).
