# Supplementary material for: Immunogenicity and Protective Efficacy of a Non-Living Anthrax Vaccine versus a Live Spore Vaccine with Simultaneous Penicillin-G Treatment in Cattle
Source: Vaccines (Basel). 2020 Oct 9;8(4):595. doi: 10.3390/vaccines8040595 (PMC7711464; doi:10.3390/vaccines8040595)
Supplement: Supplementary file 1 [file vaccines-08-00595-s001.pdf]

# Supplementary data

**Table S1.** The Anti-recombinant protective antigen (rPA) and anti-formalin inactivated spores (FIS) IgG titres ($log_{10}$) in cattle vaccinated at week 0 and 3 and measured at week0, 3 and 5 (with means and standard deviations). Cattle were vaccinated twice (week 0 and3) with purified rPA(PrPA)+FIS+Pen-G (penicillin-G), crude rPA (CrPA)+FIS+Pen-G, SLSV+FIS+Pen-G, SLSV and NegCtl+Pen G (vaccinated with Emulsigen-D®/Alhydrogel® plus Pen-G).

| Serology | Vaccine type | Week 0 | Week 3 | Week 5 |
|---|---|---|---|---|
| Anti-rPA IgG ELISA titre ($log_{10}$) | NegCtl+Pen-G | 1,25 ± 0,32 | 1,36 ± 0,34 | 1,54 ± 0,30 |
| | PrPA+FIS+Pen-G | 1,17 ± 0,29 | 3,30 ± 0,34* | 4,45 ± 0,33*** |
| | CrPA+FIS+Pen-G | 1,25 ± 0,40 | 2,93 ± 0,40* | 4,30 ± 0,75*** |
| | SLSV+Pen-G | 1,17 ± 0,22 | 1,64 ± 0,41 | 2,18 ± 0,41 |
| | SLSV | 1,22 ± 0,21 | 2,88 ± 0,38 | 4,29± 0,40*** |
| Anti-FIS IgG ELISA titre ($log_{10}$) | NegCtl+Pen-G | 1,14 ± 0,05 | 1,32 ± 0,34 | 1,53 ± 0,40 |
| | PrPA+FIS+Pen-G | 1,21 ± 0,25 | 3,36 ± 0,09* | 4,58 ± 0,36**** |
| | CrPA+FIS+Pen-G | 1,21 ± 0,29 | 3,68 ± 0,38* | 4,48 ± 0,45*** |
| | SLSV+Pen-G | 1,14 ± 0,18 | 3,11 ± 0,38* | 4,46 ± 0,40*** |
| | SLSV | 1,27 ± 0,20 | 3,37 ± 0,35* | 4,53 ± 0,29*** |
| TNA[c] titre ($NT_{50}$) ($log_{10}$) | NegCtl+Pen-G | ND | 1,38 ± 0,31 | 1,40 ± 0,36 |
| | PrPA+FIS+Pen-G | ND | 2,43 ± 0,40 | 3,46 ± 0,38*** |
| | CrPA+FIS+Pen-G | ND | 2,15 ± 0,19 | 3,30 ± 0,49*** |
| | SLSV+Pen-G | ND | 1,48 ± 0,22 | 1,56 ± 0,36 |
| | SLSV | ND | 2,26 ± 0,27 | 3,40 ± 0,43*** |

[a]Mean log10 titres ± 95% confidence interval, [b]Titres were compared to the respective pre-vaccination titres (****$p < 0.0001$, ***$p < 0.001$, **$p < 0.01$, *$p \leq$ 0.0.5), [c]TNA; Lethal toxin neutralization titres, rPA; Recombinant protective antigen, FIS; Formaldehyde inactivated spore, ND; Not detected, ns; Not significant, PrPA; Purified recombinant protective antigen, CrPA; Crude recombinant protective antigen, Pen-G: Penicillin-G, SLSV; Sterne live spore vaccine, NegCtl; Negative control

**Table S2.** The anti-recombinant protective antigen (rPA) and anti-formalin inactivate *Bacillus anthrcis 34F2* spores (FIS) titres ($log_{10}$) of different isotypes in vaccinated cattle (with means and standard deviations) of Immunoglobulin isotypes titre of vaccinated cattle measured at weeks 0, 3 (two weeks after first vaccination) and 5 (two weeks after second vaccination).

| **Serology** | **Vaccine types** | **IgM** | | | **IgG1** | | | **IgG2** | | |
|---|---|---|---|---|---|---|---|---|---|---|
| | | **Week 0** | **Week 3** | **Week 5** | **Week 0** | **Week 3** | **Week 5** | **Week 0** | **Week 3** | **Week 5** |
| **Anti-rPA Igs ELISA titre ($log_{10}$)** | NegCtl+Pen-G | 1,17±0,23 | 1,51±0,14 | 1,34±0,36 | 1,50±0,35 | 1,52±0,35 | 1,50±0,33 | 1,65±0,49 | 1,51±0,35 | 1,50±0,32 |
| | PrPA+FIS+Pen-G | 1,62±0,24 | 3,05±0,36** | 3,19±0,35** | 1,55±0,33 | 2,98±0,45* | 3,26±0,31*** | 1,50±0,29 | 2,79±0,12** | 3,39±0,48*** |
| | CrPA+FIS+Pen-G | 1,57±0,32 | 2,94±0,38** | 3,06±0,45** | 1,52±0,32 | 2,89±0,51** | 3,07±0,22** | 1,58±0,23 | 2.65±0,18* | 3,40±0,38*** |
| | SLSV+Pen-G | 1,56±0,32 | 1,70±0,28 | 1,76±0,21 | 1,51±0,28 | 1,61±0,22 | 1,83±0,30 | 1,58±0,22 | 1,69±0,77 | 1,93±0,41 |
| | SLSV | 1,64±0,23 | 2,84±0,39** | 3,02±0,45** | 1,51±0,28 | 2,81±0,27** | 2,93±0,23** | 1,54±0,24 | 2,66±0,21* | 3,56±0,32*** |
| Anti-FIS Igs ELISA titre ($log_{10}$) | NegCtl+Pen-G | 1,62±0,37 | 1,65±0,31 | 1,62±0,43 | 1,73±0,09 | 1,60±0,30 | 1,72±0,17 | 1,68±0,136 | 1,57±0,36 | 1,53±0,37 |
| | PrPA+FIS+Pen-G | 1,64±0,15 | 2,95±0,23** | 3,09±0,27** | 1,62±0,26 | 2,98±0,38** | 3,11±0,37*** | 1,61±0,23 | 2,71±0,31* | 3,24±0,32*** |
| | CrPA+FIS+Pen-G | 1,52±0,30 | 2,89±0,23** | 3,06±0,37** | 1,57±0.21 | 2,86±0,24* | 2,98±0,19** | 1,60±0,23 | 2,75±0,30* | 3,13±0,44*** |
| | SLSV+Pen-G | 1,58±0,22 | 2,74±0,23* | 2,90±0,24** | 1,62±0,21 | 1,85±0,37** | 2,29±0,46** | 1,57±0,21 | 1,97±0,30* | 2,00±0,41** |
| | SLSV | 1,51±0,27 | 2,91±0,20** | 3,08±0,27*** | 1,63±0,23 | 2,74±0,29* | 2,94±0,11** | 1,66±0,15 | 2,60±0,24* | 2,96±0,35** |

[a]Mean log10 titres ± 95% confidence interval, [b]Titres were compared to the respective pre-vaccination titres (***$p$ < 0.001, **$p$ < 0.01, *$p$ ≤ 0.0.5). rPA83; Recombinant protective antigen 83, PrPA; Purified rPA83, CrPA; Crude rPA83, Pen-G: Penicillin-G, SLSV; Sterne live spore vacci8ne, NegCtl; Negative control (Emulsigen-D®/Alhydrogel® and Pen-G).
